# Coronary Anomalies: Left Main Coronary Artery Aneurysm

**DOI:** 10.1155/2012/954951

**Published:** 2012-08-05

**Authors:** Rajsekhar Varda, Santosh Kumar Chitimilla, Aslam Lalani

**Affiliations:** Department of Cardiology, Yashoda Superspeciality Hospitals, Near Hari Hara Kala Bhavan, Clock Towers, Andhra Pradesh, Secunderabad 500003, India

## Abstract

Coronary artery aneurysm is one of the rarest anomalies that we see in our medical practice and they are mostly associated with obstructive lesions due to atherosclerotic changes. Management of these aneurysm patients (conservative or surgical repair) usually depends on obstructive lesions and associated symptoms. We are presenting a case of left main aneurysm measuring around 14 × 28 mm with other obstructive leisons. It was treated with surgical repair in view of obstructive lesions and symptoms.

## 1. Introduction

 Coronary artery aneurysm is the most rare to see in our medical practice. The first case was reported by Morgagni, while conducting postmortem study. In the order of frequency, the commonest site of aneurysm in the coronary anatomy is right coronary artery, circumflex followed by anterior descending arteries. Left main aneurysm seems to be the rarest with an incidence of 0.1%. The most common cause of coronary aneurysm mostly seems to be atherosclerotic.

## 2. Case Scenario

**Figure 1:**
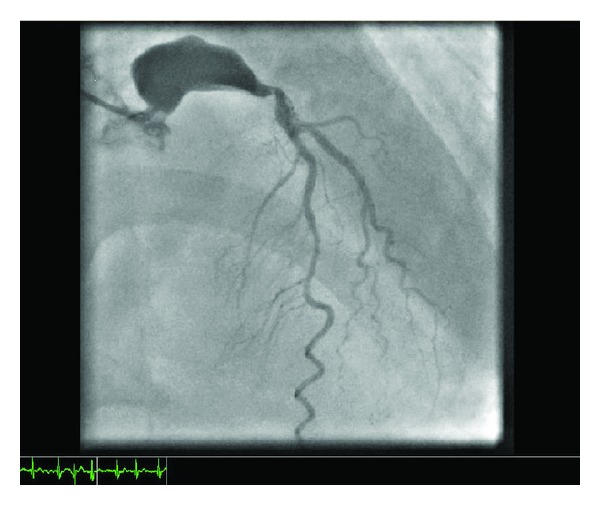
Coronary angiogram showing left main aneurysm (14 × 28) with obstructive leison at LAD ostial level.

**Figure 2:**
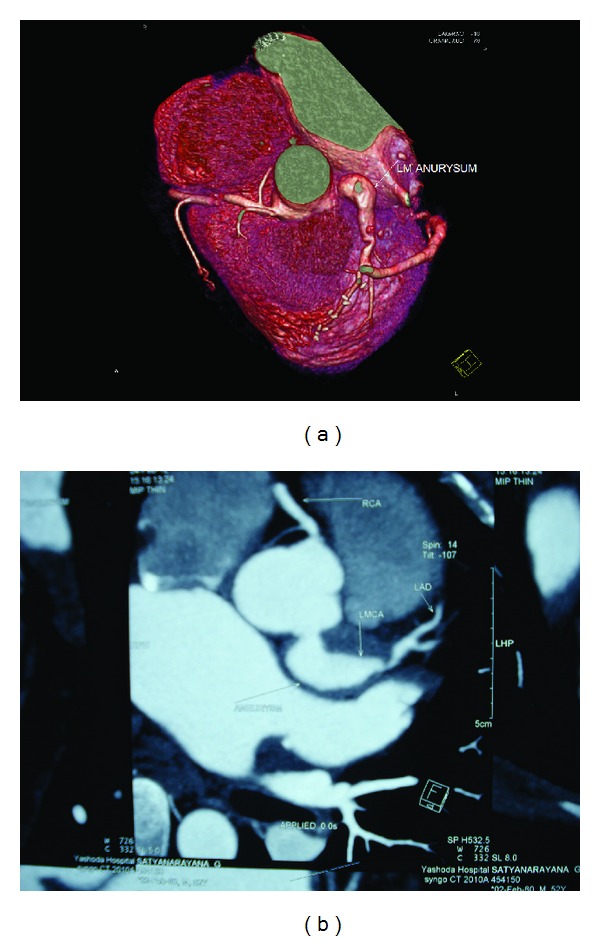
(a) CT Coronary angiogram showing left main aneurysm with ostial LAD stenosis. (b) CT Coronary angiogram showing left main aneurysm with ostial LAD stenosis.

**Figure 3:**
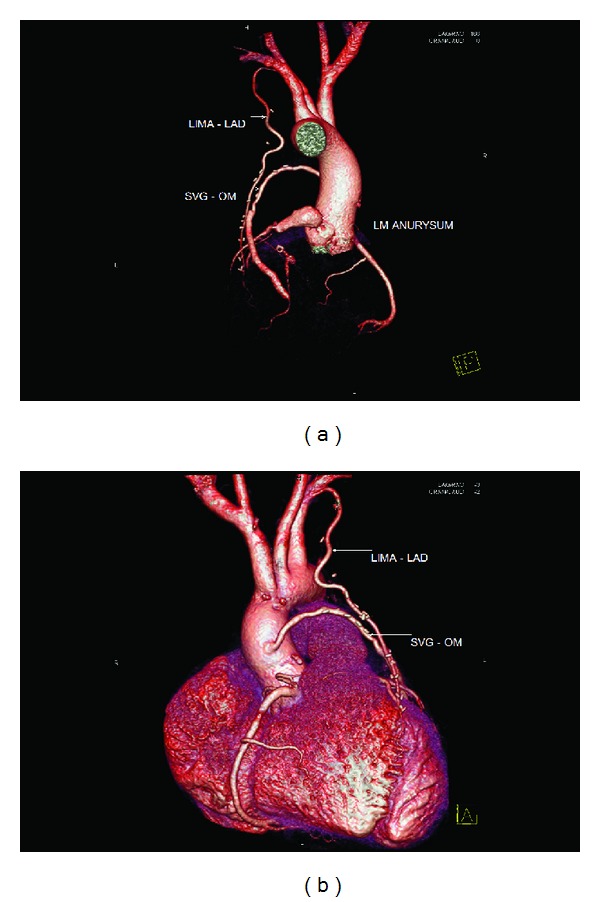
(a) Post-CABG CT Coronary angiogram showing proximally and distally ligated left main aneurysm with LIMA to LAD grafts and SVG to OM grafts. (b) Post-CABG CT Coronary angiogram showing proximally and distally ligated left main aneurysm with LIMA to LAD grafts and SVG to OM grafts.

A 52 yrs old gentleman, nonhypertensive, nondiabetic and known case of rheumatic heart disease with mitral stenosis and atrial fibrillation has presented to casualty with a history of chest pain, shortness of breath class 2-3 associated with productive cough and pyrexia for 1-2 weeks. The patient had undergone percutaneous balloon mitral valvotomy in 1999 and had undergone coronary angiogram in the same year, which revealed normal coronaries with normal left main anatomy. During preliminary investigations, his electrocardiogram was showing T-wave inversion in the precordial leads and atrial fibrillation with fast ventricular rate. His chest X-ray was showing signs of congestive heart failure. 2D-Echo revealed RWMA +ve with Hypokinetic LAD Territory, Moderate LV Dysfunction, and Tight mitral stenosis. Coronary angiogram revealed left main Aneurysm measuring 14 × 28 mm with single-vessel disease (critical ostial lesion in the LAD—Figure 1). Later on, CT Coronary angiogram (Figures 2(a) and 2(b)) has been done for further evaluation. The patient has been stabilized and then transferred to CT department for CABG (2 grafts LIMA–LAD, SVG–OM) with isolation of left main aneurysm from coronary circulation by proximal and distal ligation (Figures 3(a) and 3(b)). Simultaneously, Mitral valve replacement has been done. perioperative findings revealed mild cardiomegaly, RA, RV dilated, normal left ventricle, and atheromatous aorta.

During the postoperative stay, the patient was mechanically ventilated for 2 hours, ambulated on 2nd day, and was discharged in a haemodynamically stable condition.

## 3. Discussion

 Coronary artery aneurysms are very rare, especially left main aneurysm are even more rare [1, 2]. The prevalence rates vary from 0.25% to 2.6% [3, 4]. Coronary artery aneurysm is defined when coronary arterial segments dilates >1.5 times of normal adjacent coronary segments or largest coronary artery or three times the diameter of coronary artery catheter [5, 6].

## 4. Types of Aneurysm [7]

1. Fusiform (dilatation along the long axis of vessel at least twice the diameter of transverse dimension).
2. Saccular (transverse dimension > longitudinal dimension).

## 5. Causes of Coronary Artery Aneurysm

1. Atherosclerotic diseases [1].
2. SubAcute bacterial endocarditis.
3. Kawasaki disease.
4. Marfan syndrome.
5. Takayasus arteritis.
6. Rheumatic fever.
7. Mycosis.
8. Syphilis.
9. Trauma [8].
10. Previous balloon angioplasty [9].

Few cases have been reported—where they developed coronary artery aneurysm after drug eluting stent implantation. Usual incidence of developing aneurysm after DES implantation is 0.2–2.3%. It might be developed from 3 days to 4 years after DES stent implantation. Common risk factor for BMS & DES is mechanical risk factor. Peculiar risk factor of DES is that it causes significant inflammation of the arterial wall [10]. There are few cases reported in the literature, where giant left main aneurysm was noted without associated coronary lesion [11].

## 6. Management of Aneurysm

Indication for surgery in left main aneurysm is angina, obstructive coronary stenosis. Coronary stent graft is one of the treatment modalities for the management of left main aneurysm. Very few cases have been reported in the literature. Patients who are conservatively managed for left main aneurysm have to be on oral anticoagulation & antiplatelet therapy to prevent complications like thrombosis, embolisim, and acute MI (in the aneurismal sac, the blood flow will be sluggish) [12]. Initially, CABG alone was the surgical management for the left main aneurysm, but now along with CABG, isolation of left main aneurysm from coronary circulation is the preferred treatment modality by most of the surgeons [13]. This sort of cases, during the time of followup by 6 months to 1 year, may develop residual aneurysm [14].
